# Treatment with an anti-CD11d integrin antibody reduces neuroinflammation and improves outcome in a rat model of repeated concussion

**DOI:** 10.1186/1742-2094-10-26

**Published:** 2013-02-15

**Authors:** Sandy R Shultz, Feng Bao, Lynne C Weaver, Donald P Cain, Arthur Brown

**Affiliations:** 1 Department of Psychology and Graduate Program in Neuroscience, University of Western Ontario, London, N6A 5C2, Canada; 2 Robarts Research Institute, Schulich School of Medicine, University of Western Ontario, 100 Perth Drive St., London, Ontario, N6A 5K8, Canada

## Abstract

**Background:**

Concussions account for the majority of traumatic brain injuries (TBI) and can result in cumulative damage, neurodegeneration, and chronic neurological abnormalities. The underlying mechanisms of these detrimental effects remain poorly understood and there are presently no specific treatments for concussions. Neuroinflammation is a major contributor to secondary damage following more severe TBI, and recent findings from our laboratory suggest it may be involved in the cumulative properties of repeated concussion. We previously found that an anti-CD11d monoclonal antibody that blocks the CD11d/CD18 integrin and adhesion molecule interaction following severe experimental TBI reduces neuroinflammation, oxidative activity, and tissue damage, and improves functional recovery. As similar processes may be involved in repeated concussion, here we studied the effects of the anti-CD11d treatment in a rat model of repeated concussion.

**Methods:**

Rats were treated 2 h and 24 h after each of three repeated mild lateral fluid percussion injuries with either the CD11d antibody or an isotype-matched control antibody, 1B7. Injuries were separated by a five-day inter-injury interval. After the final treatment and either an acute (24 to 72 h post-injury) or chronic (8 weeks post-injury) recovery period had elapsed, behavioral and pathological outcomes were examined.

**Results:**

The anti-CD11d treatment reduced neutrophil and macrophage levels in the injured brain with concomitant reductions in lipid peroxidation, astrocyte activation, amyloid precursor protein accumulation, and neuronal loss. The anti-CD11d treatment also improved outcome on tasks of cognition, sensorimotor ability, and anxiety.

**Conclusions:**

These findings demonstrate that reducing inflammation after repeated mild brain injury in rats leads to improved behavioral outcomes and that the anti-CD11d treatment may be a viable therapy to improve post-concussion outcomes.

## Introduction

Concussions account for the majority of all traumatic brain injuries (TBI) and are now recognized as a serious global health concern, particularly in individuals at an increased risk of suffering concussion, such as athletes and military personnel [1-3]. Although the effects of a single concussion are often transient [3], repeated concussion has been associated with cumulative [4,5] and chronic neurological disturbances including cognitive deficits, emotional abnormalities, motor impairments, and neurodegenerative disease [6-10]. However, little is known about the factors and mechanisms that might contribute to these debilitating effects, and no specific treatment options currently exist [3,10-12]. In light of this, our laboratory has recently used repeated mild lateral fluid percussion injuries (mLFP) in the rat as a novel model of repeated concussion [5]. This work demonstrated that repeated mLFP induces cumulative long-term behavioral impairments and cortical damage consistent with those observed in the clinical population, and suggests that neuroinflammation may be associated with these effects.

Neuroinflammation is known to be a key mediator of secondary injury in moderate and severe TBI [13,14], as well as other neurodegenerative disorders [15]. The neuroinflammatory response in TBI is characterized by the activation of microglia and astrocytes, the release of pro-inflammatory cytokines and chemokines, and the infiltration of peripheral leukocytes across the blood–brain barrier and into the injured brain [14,16,17]. Infiltrating leukocytes further drive the neuroinflammatory response and exacerbate secondary injury through the production of pro-inflammatory mediators, free radicals, lipid peroxidation, and oxidative stress [14,18-20]. The infiltration of leukocytes into the CNS is mediated, in part, by CD11/CD18 integrins, a family of membrane-bound glycoproteins. The CD11/CD18 heterodimer is composed of a common CD18 subunit and one of four CD11 subunits (a to d). The CD11d/CD18 integrin is expressed on neutrophils and monocytes/macrophages, and binds to the adhesion molecule vascular cell adhesion molecule-1(VCAM-1) expressed on the surface of vascular endothelial cells in both rat and human CNS [21,22]. Previous work from our laboratory has used a CD11d monoclonal antibody (mAb) to block the CD11d/CD18-VCAM-1 interaction following spinal cord injury and after single severe fluid percussion injury in rats [18,23]. The anti-CD11d treatment reduced leukocyte levels in the injured brain with concomitant reductions in astrocyte activation, lipid peroxidation, axonal injury, and neuronal loss [18]. Further, the reduced neuroinflammation in anti-CD11d mAb-treated rats was accompanied by improved performance on behavioral tasks of cognition, anxiety-like behavior, and sensorimotor ability relative to injured rats treated with a control mAb. Since neuroinflammation may also be implicated in concussion [24], and occurs following mLFP [5,24-26], here we evaluated the effects of the CD11d mAb treatment in the repeated mLFP rat model of repeated concussion. We report that treatment with the CD11d mAb after each of three repeated mLFP reduced neutrophil and macrophage numbers, astrocyte activation, and lipid peroxidation. These reductions in mediators of secondary injury were accompanied by reductions in neuronal loss, axonal injury, and behavioral deficits.

## Materials and methods

### Subjects

All procedures and behavioral tests were in accordance with guidelines of the Canadian Council on Animal Care and approved by the University of Western Ontario Animal Use Subcommittee. Subjects were 87 young adult male Long-Evans hooded rats obtained from Charles River Laboratories (Quebec, Canada). Prior to surgery rats weighed between 250 to 300 g, and were naïve to all experimental procedures. After surgery rats were housed individually for the remainder of the study under a 12:12 light/dark cycle and were allowed access to food and water *ad libitum*.

### Treatment groups

**Figure 1:**
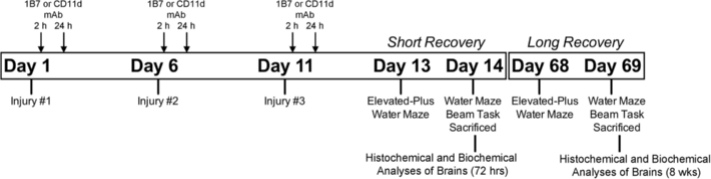
**Experimental timeline.** Rats received either three sham injuries or three mild lateral fluid percussion injuries. After each injury rats were injected with their assigned treatment at 2 h and 24 h post-injury. After the final treatment, rats had either a short- (24 h) or long- (8 weeks) recovery period before behavioral testing. After behavioral testing, rats were sacrificed for histochemical and biochemical analyses.

Rats were randomly assigned to one of three treatment groups: three sham injuries + saline treatment (sham), three mLFP + isotype-matched control mAb (1B7), and three mLFP + CD11d mAb treatment (CD11d). Rats received their assigned treatments 2 h and again 24 h after each injury via tail vein injection (1.0 mg/kg; [18]). This dosing schedule is based on the dosing schedule that we have used successfully to treat both spinal cord-injured rats and mice [27,28]. Rats had a 5-day inter-injury recovery period between repeated injuries [5]. After the final treatment, rats were designated to one of two recovery periods: short-recovery (SR, 24 hours post-injury), or long-recovery (LR, 8 weeks post-injury). Thus, there were a total of six experimental groups in the current study: sham-SR (n = 15), 1B7-SR (n = 15), CD11d-SR (n = 15), sham-LR (n = 12), 1B7-LR (n = 12), and CD11d-LR (n = 12). The timeline for the experimental protocol is outlined in Figure 1.

### Surgery and injuries

Surgeries and injuries were carried out as previously described [5,25,26]. Under anesthesia and aseptic conditions, rats underwent a craniotomy (3 mm) centered over the following coordinates with reference to Bregma: anterior/posterior −3.0 mm, medial/lateral 6.0 mm. A hollow plastic injury cap was sealed over the craniotomy and a removable plug was inserted into the injury cap to seal the craniotomy until, and after, mLFP was administered. Approximately 24 h post-surgery rats were placed under anesthetic and the rat was attached to the injury device. At the first response of hind-limb withdrawal to a toe pinch, rats in the CD11d and 1B7 groups received a single mLFP with a force of 1 to 1.5 atm. Sham-injured rats were treated identically but were removed from the injury device without receiving a mLFP. Apnea, time of unconsciousness, and self-righting reflex were all monitored immediately after each injury to assess acute injury effects. Rats received tail vein injections of the assigned treatments 2 h and 24 h after each injury. Rats were given a 5-day inter-injury recovery period before undergoing the identical injury procedure as described above for the remaining two injuries. After their final drug injection, rats were assigned to either a 24 h (SR) or 8 week (LR) recovery condition.

### Histochemical and biochemical analyses of brains

#### Tissue preparation

For histological examination at 72 h after the final injury, approximately half of the rats (n = 6 to 7 for each group) were anesthetized (2.5 g/kg urethane) and perfused transcardially with saline, followed by 4% paraformaldehyde in PBS (pH 7.2 to 7.4). Brains were removed, post-fixed for 24 h at 4°C, cryoprotected in increasing concentrations of sucrose, and sectioned into 35-μm cross-sections for immunohistochemical staining. The entire brain was sectioned coronally and floating sections containing a visible hippocampus (approximately 3 mm caudal to Bregma) were saved for immunohistochemical staining. For biochemical assays and western blotting, approximately half of the rats (n = 6 to 7 for each group) at 72 h and 8 weeks post-injury were perfused with cold 0.9% NaCl and their brains removed. The brainstem and cerebellum were removed and the middle third of the injured hemisphere homogenized and stored at −80°C [18].

#### Immunohistochemistry and histology

Monoclonal mouse antibodies, anti-ED1 (1:500, Serotec, Raleigh, NC, USA), anti-NeuN (1:500, EMD Millipore Corporation, Billerica, MA, USA), anti-Glial Fibrillary Acidic Protein (GFAP, 1:500, Sigma, St. Louis, MO, USA) and anti-APP (1:200, Sigma), and polyclonal rabbit anti-neutrophil antibody (1:20000, gift of Dr. Daniel Anthony, Oxford University, Oxford, UK), were used for immunohistochemical staining. Immunoreactivity was revealed with a glucose-diaminobenzidine-nickel solution. Representative sections of the injured area from each animal were processed free-floating for staining as described previously [18]. Fields of view were captured digitally by a Retiga 1300 camera (Quantitative Imaging Corporation, Burnaby, BC, Canada), and ImagePro Plus Software (Media Cybernetics, Silver Spring, MD, USA). Photomicrographs were captured from coronal sections deemed by inspection of the tissue structure to be at the site of injury. A 20× field of view of the cortex above the hippocampus was sampled. Cells were counted visually and an average number from three sections was calculated for each animal. Because sections were sampled from a group of floating sections, stereological methods for counting were not used.

Neutrophil, macrophage and neuronal cell counts in shams (n = 5), 1B7 controls (n = 4) and anti-CD11d-treated (n = 4) rats at 72 h after the third injury were done by counting the number of cells immunoreactive for the anti-neutrophil, anti-ED1 and anti-NeuN antibodies, respectively. The analyses were done blinded to the injury or treatment of the rats. The 20× fields of view selected for counting neutrophils and macrophages were those with the greatest density of inflammatory cells. Approximately the same field of view on a different section in each animal was used to count cells immunoreactive for NeuN. Using a similar protocol the area of GFAP immunoreactivity was also quantified in these rats at the same time point. In three sections, fields of view (20×) were again selected that approximated the same region analyzed for inflammatory cells and area per area measurements of GFAP immunoreactivity were obtained and averaged. When no cortical injury or inflammatory cells were apparent, fields of view were selected from the same location as in the other rats.

#### Western blotting analysis

Tissue sample preparation and protein determination were performed as described previously [18]. Primary antibodies used included: anti-ED1 (1:1000, Serotec), anti-GFAP (1:5000, Sigma), anti-neutrophil (1:20000), anti-NeuN (1:5000, EMD Millipore), anti-APP (1:2000, Sigma) and anti-β-actin (1:10000, Sigma). Signal detection was facilitated with enhanced chemiluminescence (ECL kit, Amersham, Piscataway, NJ, USA). Immunoreactive bands were scanned by an imaging densitometer (BioRad GS-700 Imaging Densitometer) and the results were quantified using Multi-Analyst software (Bio-Rad). Densitometric values were normalized for protein loading using β-actin as a loading control [18].

#### Myeloperoxidase (MPO) activity assay

MPO enzymatic activity is derived mostly from neutrophils and to a lesser extent from macrophages. This activity, an estimate of the extent of neutrophil infiltration/activation and macrophage activation, was measured as described previously [18]. MPO activity was calculated using a standard curve prepared with purified human MPO (Sigma). Results are expressed as units per mg protein.

#### Measurement of lipid peroxidation

The relative levels of aldehydes including malondialdehyde (MDA), indicators of lipid peroxidation, were measured using thiobarbituric acid reactive substances (TBARS) assay as described previously [18,29]. MDA bis (dimethyl acetal; Sigma) was used as a standard, and the level of lipid peroxide was expressed as nmol of TBARS.

### Behavioral testing

#### Water maze

Behavioral testing spanned 2 days, beginning 24 h after the final treatment for SR rats and 8 weeks after the final treatment for LR rats. Spatial cognition was assessed using a water maze as described previously [18,25]. Acquisition training consisted of 10 trials with a maximum time of 60 s and occurred immediately after elevated-plus maze testing. Reversal training was carried out 24 h after acquisition and consisted of 10 trials with the hidden escape platform located in the opposite quadrant of the acquisition session. Water maze behavior was analyzed by a tracking system that digitized each swim trial (Poly-Track, San Diego Instruments, San Diego, CA, USA). Search latency and direct and circle swims were used as measures of spatial place memory in the water maze. Swim speed was used as a measure of motor ability. For graphic presentation of search time data in Results, the time to reach the platform was averaged for every block of two trials (for example, block 1 = (trial 1 + trial 2)/2).

#### Elevated plus maze

Anxiety-like behavior was assessed using an elevated-plus maze as described previously [18,25]. Rats were first placed in the center of the elevated-plus maze facing an open arm and allowed to explore the maze freely for 5 minutes. Following testing, a videotape of the trial was scored by a person blind to treatment group. As time spent in the open arm is decreased in rats that exhibit greater anxiety-related behaviors, a percentage score was calculated for the time spent in the open arm as follows: time in the open arm/(time in the open arm + time closed arm) [25,30]. The number of closed arm entries was also calculated as a measure of locomotion [31,32]. All four paws had to enter an arm for it to be considered an entry.

#### Beam task

After the completion of water maze reversal testing, sensorimotor ability was evaluated using a narrow wooden beam 1 m long and 2 cm wide as described previously [18,25]. A video camera recorded all trials. After testing, the videotape was scored by an individual who was blind to group membership to determine traverse times and the number of slips and falls. Traverse time was defined as the time required to cross the 1 m long beam, with a maximum allowed time of 60 s. Slips and falls were scored when any limb slipped from the beam, or the rat fell off the beam. Rats that fell from the beam were given a maximum time of 60 s.

#### Statistical analyses

Water maze search time and beam traverse time data were analyzed by SPSS 17.0 using mixed design analysis of variance (ANOVA) with treatment group as the between-subjects factor and trial as the within-subjects factor. One-way ANOVA, with treatment group as the between-subjects factor, were used to analyze acute injury measures, percent of time in open arm, closed-arm entries, direct and circle swims, swim speed, and slips and falls. Fisher’s least significant difference (LSD) post hoc pair-wise comparisons were carried out on behavioral data when appropriate. Histochemical and biochemical results were analyzed using randomized one-way ANOVA. Student Neuman Keuls post hoc tests were carried out for these analyses when appropriate. Statistical significance was set at *P ≤*0.05. Throughout the Results, the values from the ANOVA are presented in the text. Statements of differences between mean values and indications of significance on the figures assume *P*-values ≤0.05 in the post hoc tests.

## Results

### Acute injury measures

**Figure 2:**
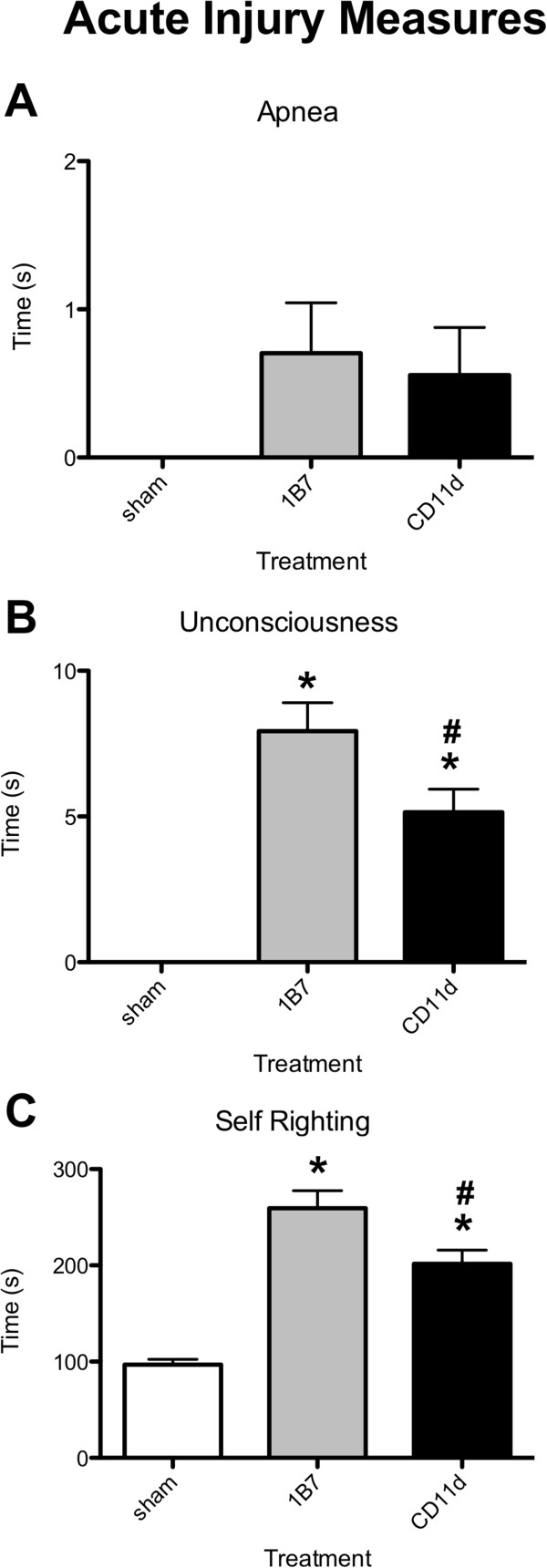
**Anti-CD11d treatment improves recovery in the acute period after repeated mild fluid percussion injury.** Treatment group of rats in panels A-C is indicated by the legend in panel A (n = 27/group). (**A**) Plot of duration of apnea following the final (third) injury. (**B**) Plot of duration of unconsciousness following the final injury. (**C**) Plot of time required for self-righting following the final injury. Histogram bars represent means (± standard error of the mean). *Different from sham-injured group; #different from 1B7 group. For post hoc comparisons of means, all *P* ≤0.05. For additional statistical details see Results.

Measures of the rats’ acute recovery from the mLFP were analyzed statistically after the final assigned (third) injury. The duration of apnea after mLFP did not differ between groups (*F*_2,78_ = 1.879, *P =* 0.160) (Figure 2A). The duration of unconsciousness (*F*_2,78_ = 30.762, *P <*0.001) (Figure 2B) and time required for self-righting (*F*_2,78_ = 36.143) *P <*0.001) (Figure 2C) were significantly increased in both the 1B7 and CD11d groups compared to the sham-injured group. Treatment with the CD11d mAb significantly improved the rats’ acute recovery as the CD11d group had shorter periods of unconsciousness and self-righting reflex times than the 1B7 group.

### Histochemical and biochemical findings at 72 h and 8 weeks after repeated mild fluid percussion injury

#### Neutrophils

**Figure 3:**
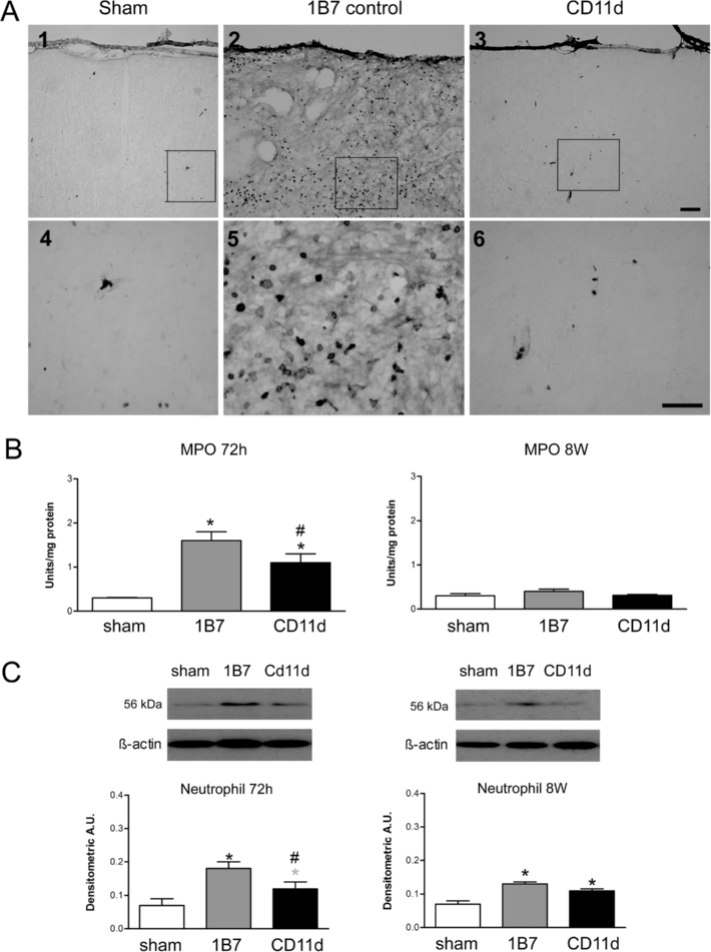
**Neutrophil infiltration after repeated mild fluid percussion injury is reduced by the anti-CD11d treatment.** (**A**) Photomicrographs of brain sections at the cortical injury site, or a comparable site in the sham-injured rats, immunostained by an anti-neutrophil antibody. The sections from sham-injured, 1B7 and anti-CD11d-treated rats at 72 h after injury are shown at lower power in panels 1 to 3 and higher power in panels 4 to 6. Scale bars = 100 μm. Scale bar in panel 3 refers to panels 1 to 3. Scale bar in panel 6 refers to panels 4 to 6. (**B**) MPO activity in brain homogenates in sham-injured groups (n = 6, both times), 1B7 groups (n = 7 and 6, respectively), and CD11d groups (n = 6 and 5 respectively) at 72 h and at 8 weeks post-injury. A.U., arbitrary units; *different from sham-injured group; #different from 1B7 group; all *P* ≤0.05 except that the gray *indicates *P* = 0.067. (**C**). Neutrophil protein, identified by western blotting in brain homogenates expressed as mean values ± standard error of the mean, with a representative autoradiogram shown above each set of histograms (n = 6 for each group).

We assessed the effect of the anti-CD11d treatment on neutrophil infiltration at 72 h and 8 weeks post-injury by immunostaining brain sections with an antibody to a 56 kDa protein expressed by neutrophils [18,33]. Sham brains contained very few neutrophils (Figure 3A, panels 1 and 4), whereas many neutrophils were detected near the repeated mLFP site in the cortex of the 1B7-treated rats (Figure 3A, panels 2 and 5). The density of these cells appeared reduced in the anti-CD11d-treated rats, (Figure 3A, panels 3 and 6). An indirect measure of neutrophil density in the brain was quantified using the anti-neutrophil antibody in a western blot analysis (Figure 3B). At 72 h post-injury, MPO activity was low in homogenates from sham-injured rats but significantly increased in the two repeated mLFP groups (F_2,16_ = 15.265, *P* <0.001). The neutrophil protein expression in the brain homogenates of the CD11d mAb-treated rats at this time was significantly less than in the 1B7 mAb-treated controls (F_2,15_ = 10.209, *P* <0.01). Direct neutrophil cell counts (anti-neutrophil immunoreactive cells) confirmed these findings revealing an average of 2.3 ± 1.2, 185.0 ± 77.0 and 2.3 ± 1.0 neutrophils per 20× field located at the lesion in shams, 1B7 controls and anti-CD11d-treated rats, respectively. The differences in cell counts between the CD11d- and 1B7-treated groups and between the shams and the 1B7 group were statistically significant (F_2,10_ = 6.48, *P* <0.05, Student Neuman Keul test, *P* <0.05). At 8 weeks post-injury, neutrophil protein expression was not significantly different between CD11d- and 1B7-mAb-treated rats.

The effect of the anti-CD11d treatment was also assessed on MPO enzymatic activity, a measure of neutrophil infiltration/activation and, to a lesser degree, of macrophage activation (Figure 3C). At 72 h post-injury, MPO activity was low in homogenates from sham-injured rats but significantly increased in the two repeated mLFP groups (F_2,16_ = 15.265, *P* <0.001). Anti-CD11d-treated rats had significantly less MPO activity compared to the control 1B7-treated rats. MPO activity was minimal in all groups at 8 weeks post-injury.

#### Microglia/macrophages

**Figure 4:**
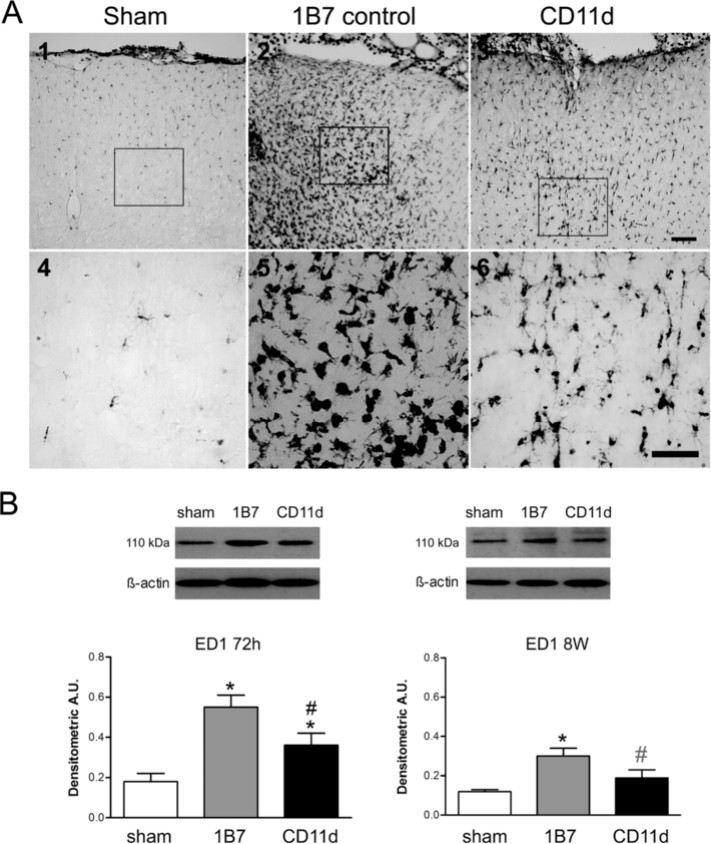
**Macrophages in the brain after repeated mild fluid percussion injury are reduced by the anti-CD11d treatment.** (**A**) Photomicrographs of brain sections at the injury or equivalent site at 72 h after injury in the cortex in sham-injured, 1B7 control and anti-CD11d-treated rats immunostained by an anti-ED1 antibody. Format is similar to that described for Figure 3. Scale bars = 100 μm. (**B**) Macrophage protein, identified by western blotting in brain homogenates expressed as mean values (± standard error of the mean) with a representative autoradiogram shown above each set of histograms. Macrophage protein is shown at 72 h and 8 weeks in sham-injured, 1B7 and anti-CD11d groups (n = 6 all groups). A.U., arbitrary units; *different from sham group; #different from 1B7 group; all *P* ≤ 0.05 with the exception that gray # indicates *P* = 0.073.

The effect of the anti-CD11d treatment on microglia/macrophage populations was assessed at 72 h and 8 weeks post-injury by immunostaining brain sections with an antibody to ED-1 (Figure 4). Only a few ED-1 immunoreactive cells were present in the brains of sham-injured rats (Figure 4A, panels 1 and 4). The size and number of ED-1 cells was clearly increased in the 1B7 rats (Figure 4A, panels 2 and 5). These cells had the rounded morphology typical of macrophages and activated microglia and they were distributed from the superficial cortical layers through to the deeper layers. In the brains of CD11d mAb-treated rats, the density of the ED-1 macrophages appeared reduced (Figure 4A, panels 3 and 6). The presence of microglia/macrophages was quantified indirectly using western blot analysis for ED-1 (Figure 4B). At 72 h post-injury, ED-1 expression (110 kDa band) was low in the brain homogenates of sham-injured rats but increased significantly in the two repeated mLFP groups (F_2,15_ = 10.769, *P* = 0.001). Direct macrophage cell counts (anti-ED1 immunoreactive cells) confirmed these findings revealing an average of 28.5 ± 10.7, 343.0 ± 73.7 and 43.4 ± 5.8 macrophages per 20× field located at the lesion in shams, 1B7 controls and anti-CD11d-treated rats, respectively. The differences in cell counts between the CD11d- and 1B7-treated groups and between the shams and the 1B7 group were statistically significant (F_2,10_ = 19.88, *P* <0.001, Student Neuman-Keuls test, *P* <0.05). At 8 weeks post-injury, ED-1 expression increased only in the brain homogenates of the 1B7-treated rats. ED-1 expression in anti-CD11d-treated rats did not differ from shams and tended to be lower than that of the 1B7-treated controls (*P =* 0.073).

#### GFAP

**Figure 5:**
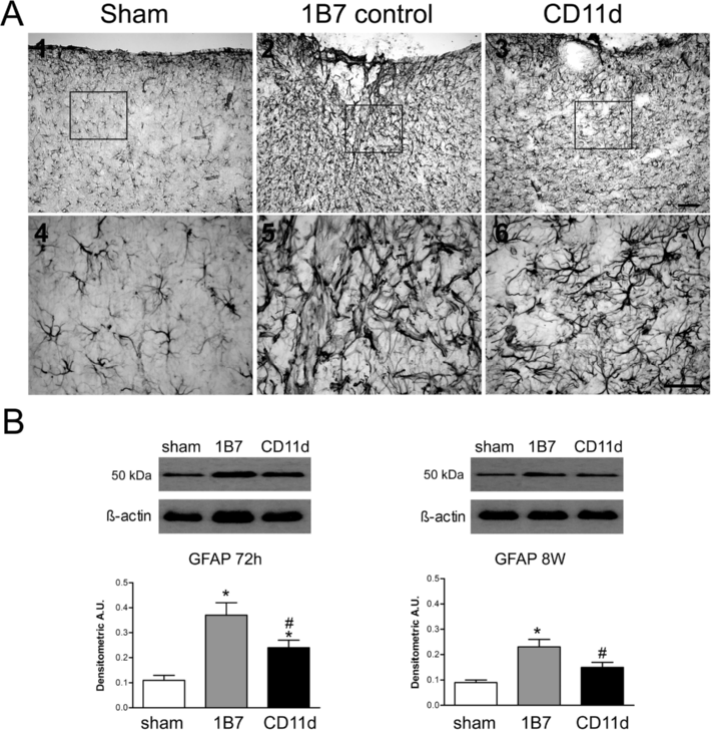
**Astrogliosis assessed by the expression of glial fibrillary acidic protein (GFAP) in the brain after repeated mild fluid percussion injury is reduced by the anti-CD11d treatment.** (**A**) Photomicrographs of brain sections at the location of cortical injury or equivalent site, at 72 h after injury, immunostained by an antibody to GFAP. Format is as in Figure 3. Scale bars = 100 μm. (**B**) GFAP, identified by western blotting in brain homogenates, shown at 72 h and 8 weeks in sham-injured, 1B7, and anti-CD11d groups (n = 6, all groups). A.U., arbitrary units; *different from sham-injured group; #different from 1B7 group; all *P* ≤0.05.

The effect of the anti-CD11d treatment on astrocyte activation in the sham-injured, 1B7, and CD11d rats was assessed by immunohistochemistry using the GFAP antibody on brain sections (Figure 5). GFAP staining demonstrated increased astrogliosis with densely stained astrocytes bearing long, overlapping hypertrophic processes in the brains of the 1B7 rats (Figure 5A, panels 2 and 5) compared to similar sections from CD11d-treated or sham-injured rats (Figure 5A panels 1, 3, 4, and 6). GFAP levels were quantified by western blot analysis (Figure 5B). GFAP (approximately 50 kDa) expression was significantly lower in CD11d-treated rats compared to 1B7-treated rats at 72 h and 8 weeks post-injury (72 h: F_2,15_ = 14.144, *P* <0.001; 8 weeks: F_2,15_ = 8.02, *P <*0.01). Evaluating the area of GFAP immunoreactivity at the lesion epicenter confirmed the western blot findings at 72 h revealing an average area of immunoreactivity of 0.012 ± 0.001, 0.22 ± 0.03 and 0.15 ± 0.02 percent (as percent of total area in a 20× field located at the lesion) in shams, 1B7 controls and anti-CD11d-treated rats, respectively. The differences in percent area of GFAP immunoreactivity between the CD11d- and 1B7-treated groups and between the shams and the 1B7 group were statistically significant (F_2,10_ = 30.47, *P* <0.001, Student Neuman-Keuls test, *P* <0.05). At 8 weeks post-injury GFAP levels as determined by western blot analyses in the CD11d-treated rats were not different from shams.

#### Neuronal survival

**Figure 6:**
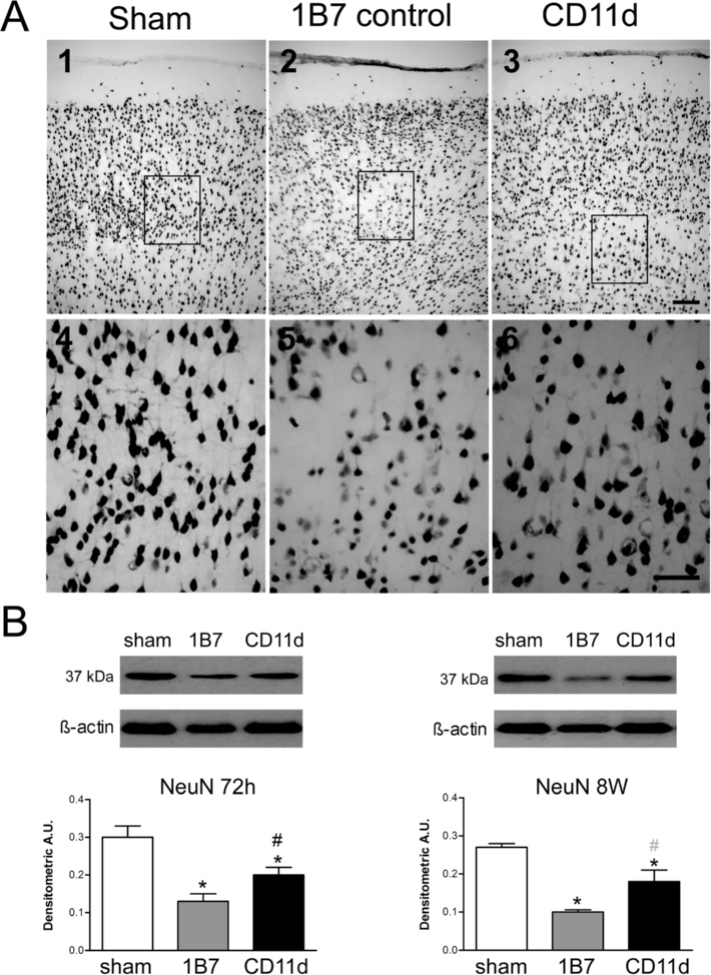
**Neuronal loss assessed by expression of the neuronal protein NeuN in the brain after repeated mild fluid percussion injury is decreased by the anti-CD11d treatment. **(**A**) Photomicrographs of brain sections at the cortical injury site or equivalent region, at 72 h after injury, immunostained by an antibody to NeuN. Format is as in Figure 3. Scale bars = 100 μm. (**B**) NeuN, identified by western blotting in brain homogenates, is shown at 72 h and 8 weeks in sham-injured, 1B7, and anti-CD11d groups (n = 6, all groups). A.U., arbitrary units; *different from sham-injured group; #different from 1B7 group; all *P* ≤0.05 with the exception that gray # indicates *P* = 0.066.

The effect of the anti-CD11d treatment on neuronal survival was assessed by immunostaining with an antibody to NeuN (Figure 6). NeuN staining in the cortex of sham brains was dense (Figure 6A, panels 1 and 4), whereas staining was less intense near the cortical injury site in 1B7-treated rats (Figure 6A, panels 2 and 5). After anti-CD11d treatment, the density of these cells appeared more like that in the sham-injured rats (Figure 6A, panels 3 and 6). To provide an indirect quantification of neuronal survival, western blotting for the marker NeuN was done using the brain homogenates (Figure 6B). At 72 h post-injury, densitometry demonstrated that although both injured groups had lower levels of NeuN than sham-injured rats, the anti-CD11d mAb-treated rats had greater levels of NeuN expression than the 1B7 controls (F_2,15_ = 11.009, *P* = 0.001). Direct neuronal cell counts (anti-NeuN immunoreactive cells) confirmed these findings revealing an average of 409 ± 22.1, 155 ± 93.3 and 382 ± 13.2 neurons per 20× field located at the lesion in shams, 1B7 controls and anti-CD11d-treated rats, respectively. The differences in cell counts between the CD11d- and 1B7-treated groups and between the shams and the 1B7 group were statistically significant (F_2,10_ = 7.07, *P* <0.05, Student Neuman-Keuls test, *P* <0.05). At 8 weeks post-injury, NeuN expression decreased significantly in both repeated mLFP groups (F_2,15_ = 9.142, *P* <0.01). However, a non-significant trend suggested that the NeuN expression in anti-CD11d-treated rats was greater than that in the 1B7 controls (*P =* 0.066).

#### Amyloid precursor protein (APP)

**Figure 7:**
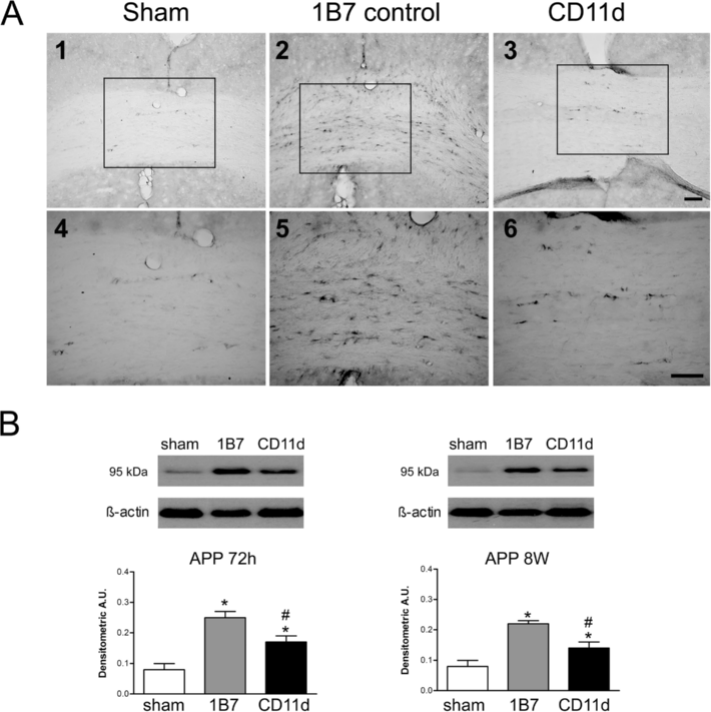
**White matter injury measured by the increase in amyloid precursor protein (APP) expression in the brain after repeated mild fluid percussion injury is reduced by the anti-CD11d treatment.** (**A**) Photomicrographs of brain sections at the center of the corpus callosum, 72 h after injury, immunostained by an antibody to APP. Format is as in Figure 3. Scale bars = 100 μm. (**B**) APP, identified by western blotting in brain homogenates, is shown at 72 h and 8 weeks in sham-injured, 1B7, and anti-CD11d groups (n = 6, all groups). A.U., arbitrary units; *different from sham-injured group; #different from 1B7 group; all *P* ≤0.05.

APP accumulation in white matter after TBI is an indicator of axonal injury [25,34]. As the corpus callosum is commonly affected by TBI [34], and contains a high density of intercortical axon projections, the effect of the anti-CD11d treatment on APP accumulation in the corpus callosum was assessed by immunostaining with an antibody to APP (Figure 7). APP accumulated minimally in the brains of sham-injured rats (Figure 7A, panels 1 and 4) but was clearly increased in the 1B7 control rats (Figure 7A panels 2 and 5). APP appeared to accumulate to a lesser extent in the brains of anti-CD11d-treated rats (Figure 7A panels 3 and 6). APP expression was quantified in rat brain homogenates using western blot analysis (Figure 7B). APP expression (90 kDa) increased significantly with repeated mLFP at 72 h (F_2,15_ = 17.828, *P* <0.001) and 8 weeks post-injury (F_2,15_ = 17.629, *P* <0.001). However, at both time points, APP expression was lower in the anti-CD11d treatment groups than in the 1B7 controls.

#### Lipid peroxidation

An indication of lipid peroxidation due to free radical tissue damage and/or to activation of the arachidonic acid cascade was evaluated by a TBARS assay that measures the relative levels of MDA and other aldehydes in tissue homogenates. At 72 h post-injury, lipid peroxidation in the brain homogenates of the CD11d mAb-treated rats (33.3 ± 3 nmol/mg protein) was significantly less than in the brain homogenates of the 1B7 mAb-treated controls (56.1 ± 4 nmol/mg protein) and not different from the sham-injured rats (34.2 ± 3.3 nmol/mg protein) (F_2,16_ = 14.072, *P* <0.001, Student Neuman-Keuls test, *P* <0.05). By 8 weeks post-injury, lipid peroxidation was increased in both repeated mLFP groups above values in sham-injured rats (F_2,14_ = 6.658, *P* <0.01, Student Neuman-Keuls test, *P* <0.05).

### Behavioral findings after repeated mild fluid percussion injury

#### Water maze

**Figure 8:**
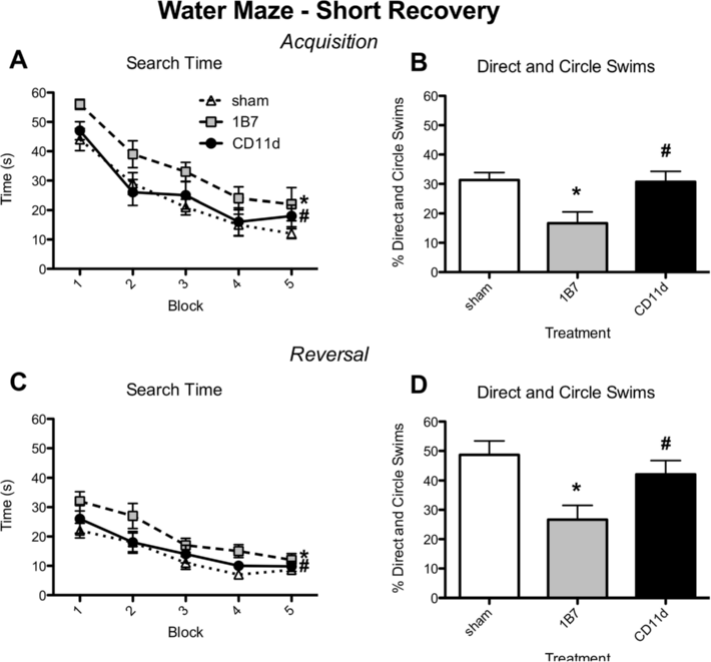
**Anti-CD11d treatment improves cognitive performance in the water maze in the short recovery period (24 h) after repeated mild fluid percussion injury.** Treatment groups for panels A-D are defined in the legend in panel A (n = 15/group). (**A**) Plot of search time vs. sequential trial during acquisition training in the water maze test. Each block is the average of two trials for each rat and data points are mean ± standard error of the mean (SEM) for the group of rats. (**B**) Plot of percent of direct and circle swims during acquisition training. Histogram bars represent means of data for the groups of rats collected during the 10 trials (± SEM). (**C**) Plot of search time with respect to number of trials (as in A) during reversal training. (**D**) Plot of percent of direct and circle swims during reversal training. *Different from sham-injured group; #different from 1B7 group; for post hoc comparisons of means, all *P* ≤0.05. For additional statistical details see Results.

Search time and the percentage of direct and circle swims in the water maze were used as measures of spatial cognition. Swim speed was used as a measure of locomotion. In the SR condition, both acquisition and reversal search times decreased in all groups as testing progressed (acquisition: trial *F*_9,369_ = 22.034, *P <*0.001, Figure 8A; reversal: *trial F*_9,369_ = 13.799, *P <*0.001, Figure 8C). However, the CD11d rats had shorter search times than the 1B7 rats and did not differ from the sham group, whereas the 1B7 rats had longer search times than shams (acquisition: treatment *F*_2,43_ = 5.795, *P <*0.01, Figure 8A; reversal: treatment *F*_2,43_ = 6.016, *P <*0.01, Figure 8C). The SR direct and circle swim data were consistent with the search time data. They revealed more direct and circle swims in the CD11d group than in the 1B7 group during both acquisition and reversal, no difference between the CD11d rats and shams, and significantly fewer direct and circle swims in the 1B7 group than in sham-injured rats (acquisition: *F*_2,43_ = 6.161, *P <*0.01, Figure 8B; reversal: *F*_2,43_ = 5.727, *P <*0.01, Figure 8D). Swim speed did not differ between the groups (all *P >*0.05; data not shown).

**Figure 9:**
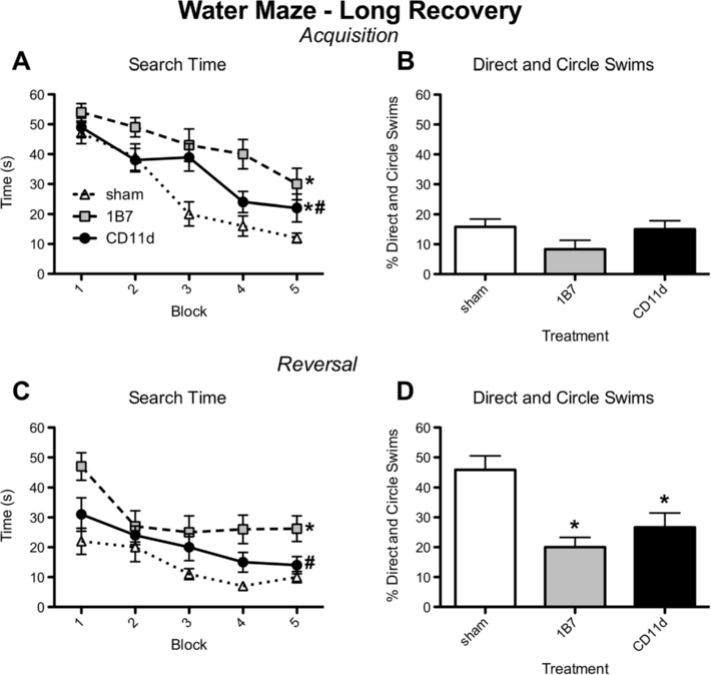
**Anti-CD11d treatment improves cognitive performance in water maze in the long recovery period (8 weeks) after repeated mild fluid percussion injury.** Format of figure is identical to that of Figure 8 (n = 12/group). (**A**) Plot of search time vs. sequential trial during acquisition training in the water maze test. (**B**) Plot of percent of direct and circle swims during acquisition training. (**C**) Plot of search time with respect to number of trials (as in A) during reversal training. (**D**) Plot of percent of direct and circle swims during reversal training. *Different from sham-injured group; #different from 1B7 group; for post hoc comparisons of means, all *P* ≤0.05. For additional statistical details see Results.

In the LR condition both acquisition and reversal search times decreased in all groups as testing progressed (acquisition: trial *F*_9,297_ = 14.962, *P <*0.001, Figure 9A; reversal: trial *F*_9,297_ = 7.198, *P <*0.001; Figure 9C). However, the CD11d rats had shorter search times than the 1B7 rats, although both remained different from shams (acquisition: treatment *F*_2,33_ = 9.057, *P <*0.001, Figure 9A; reversal: treatment *F*_2,33_ = 7.415, *P <*0.01, Figure 9C). No significant group differences in direct and circle swims were detected between the groups during acquisition (*P >*0.05; Figure 9B), whereas both CD11d and 1B7 rats had fewer direct and circle swims compared to sham-injured rats during reversal (*F*_2,35_ = 9.680, *P <*0.001, Figure 9D). The groups did not differ with respect to swim speed (*P >*0.05; data not shown).

#### Elevated-plus maze

Time spent in the open arms of the elevated-plus maze was used to measure anxiety-like behavior. The number of closed arm entries was used as a measure of locomotion. During both SR and LR elevated-plus maze testing the 1B7 group, but not the CD11d group, spent less time in the open arms compared to the sham group (SR: *F*_2,43_ = 3.283, *P <*0.05; LR: *F*_2,35_ = 3.459, *P <*0.05, data not shown). The number of closed arm entries did not differ between groups (all *P >*0.05, data not shown).

#### Beam task

Traverse time and the number of slips and falls were used as measures of sensorimotor ability on the beam task. The SR groups did not differ in traverse times (*P >*0.05; data not shown) or in the measure of slips and falls (*P >*0.05; data not shown). However, in the LR condition the 1B7 group, but not the CD11d group, displayed significantly more slips and falls than the sham group (*F*_2,35_ = 4.861, *P* <0.05, data not shown).

## Discussion

### Neuroinflammation and tissue damage

Consistent with previous findings from our laboratory [5], repeated mLFP in the rat induced a long-term neuroinflammatory response involving increases in activated neutrophils, microglia/macrophages, and astrocytes within the injured brain. This response occurred concurrently with increased lipid peroxidation, axonal injury, neuronal loss, and behavioral impairments. The oxidative burst of activated neutrophils and macrophages generates free radicals that can lead to secondary CNS injury in the form of peroxidation of lipid membranes [19,35,36]. The enzyme MPO is part of the pathway that generates oxidative stress via formation of hydrogen peroxide and other oxidative molecules, and is present in large quantities in granules within neutrophils and, to a lesser extent, macrophages [37,38]. Activated astrocytes are also active participants in neuroinflammation [39]. Astrocytes respond to a host of pro-inflammatory mediators released by activated leukocytes, and in turn release additional cytokines that can regulate microglia, neurons, and other astrocytes [40].

Anti-CD11d mAb treatment significantly reduced activated neutrophils, macrophages, and astrocytes in the SR condition relative to repeated mLFP rats treated with a 1B7 control mAb. Analysis of neuronal loss, as monitored by immunohistochemistry and western blot analysis of brain homogenates, revealed a significant increase in NeuN expression in the CD11d-SR rats compared to the 1B7-SR rats. Additionally, analysis of axonal injury, as monitored by immunohistochemical and western blot analysis of the corpus callosum, revealed a significant decrease in APP accumulation in the CD11d-SR rats compared to the 1B7-SR rats. Reductions in neuronal loss and axonal injury by anti-CD11d mAb are both likely mechanisms for the behavioral recovery associated with the treatment. As the anti-CD11d mAb treatment also reduced MPO levels and lipid peroxidation in the injured brain, oxidative stress and lipid peroxidation appear to be key mechanisms of inflammatory-mediated secondary damage and associated behavioral impairments in repeated mLFP. We have previously reported that the anti-CD11d treatment changes the nature of the cytokine/chemokine response from a pro-inflammatory response to a wound healing response when delivered acutely after spinal cord injury [41]. Thus the reduced recruitment of neutrophils and macrophages to the lesion site may have led to altered levels of cytokine and chemokine production at the lesion and consequently to reduced levels of leukocyte activation and immune-mediated tissue damage.

At 8 weeks post-injury, the anti-CD11d mAb-treated rats continued to exhibit significant reductions in astrogliosis, axonal injury, and behavioral impairments relative to 1B7-treated rats. However, the anti-CD11d mAb-treated rats were impaired relative to sham controls on some behavioral measures, and exhibited significant neuronal loss, axonal injury, and lipid peroxidation. As the anti-CD11d treatment reduced but did not completely prevent neutrophil and macrophage infiltration of the injured brain, a small inflammatory response may have led to these effects. As the current study is the first to use the anti-CD11d mAb in the complex pathophysiological setting of repeated concussion, the treatment regimen applied may not have been optimal, generating a less robust outcome than may be possible. Other mechanisms of secondary injury are also likely to contribute to the pathology of repeated concussion and might account for some of the detrimental effects observed here [10]. Nonetheless, anti-CD11d mAb treatment at 2 h and 24 h after each of three mLFP significantly reduced neuroinflammation, lipid peroxidation, neuronal loss, axonal injury, and behavioral impairments, indicating that neuroinflammation may be a key contributor to secondary damage in repeated concussions and associated neurodegenerative conditions such as chronic traumatic encephalopathy.

### Behavioral outcome

The anti-CD11d mAb treatment improved cognitive function following repeated concussion, as CD11d rats outperformed 1B7 rats in the water maze. During the SR condition, the CD11d rats displayed no impairments in the water maze compared to sham-injured rats and performed better than the 1B7 rats on all measures. Consistent with previous findings from our laboratory, long-term cognitive impairments were present in rats with repeated concussion at 8 weeks post-injury relative to sham controls [5], regardless of treatment. However, the CD11d rats continued to outperform the 1B7 rats, requiring significantly less time to locate the hidden platform during both acquisition and reversal compared to the 1B7 group.

The 1B7-treated rats also displayed a significant increase in anxiety-like behavior in both the SR and LR conditions as they spent significantly less time in the open arm of the elevated-plus maze compared to the sham controls [32,42], while the CD11d-treated groups did not [5]. These findings are consistent with previous findings from our laboratory indicating that repeated mLFP increase anxiety-like behavior, and that the anti-CD11d mAb treatment reduces the development of anxiety in rats after TBI [18].

The 1B7-LR group also exhibited significantly more slips and falls on the beam task compared to sham-LR rats, while the CD11d-LR group did not. These findings are consistent with previous findings from our laboratory indicating that anti-CD11d mAb treatment preserves motor function following TBI [18]. The finding that the 1B7 group displayed deficits on the beam task in the LR condition suggests that locomotor ability should be considered when interpreting water maze and elevated-plus maze results. However, although 1B7 rats displayed increased slips and falls in the LR condition, they were not impaired on the measures of beam traverse times, swim speed in the water maze, and the number of closed arm entries in the elevated-plus maze, suggesting that the impairments suffered by 1B7 rats represent a fine motor or gait deficit rather than a gross motor abnormality [43]. Furthermore, impairments in the water maze and elevated-plus maze also occurred in the 1B7-SR group in the absence of any evidence of motor abnormalities. Thus, it appears unlikely that any motor impairments suffered by the 1B7 group directly accounted for the water maze and the elevated-plus findings.

These results are similar to the findings of other studies in which antibody treatments targeted the infiltration of peripheral leukocytes after single severe TBI in rats [18,23,44,45]. Specifically, we have previously reported that anti-CD11d mAb treatment reduced leukocyte levels, astrocyte activation, lipid peroxidation, and neuronal loss in a severely injured brain, and these changes were accompanied by improved cognitive, emotional, and sensorimotor outcomes relative to injured rats treated with a control mAb [18,23]. Although key methodological differences limit direct comparisons between these experiments and the current study, it is important to note that anti-CD11d mAb therapy had similar benefits in both mild and severe TBI settings. Furthermore, despite the overall reduction of neutrophil and macrophage populations in the injured brains, animals treated with anti-CD11d mAb do not appear to suffer infections in the CNS or elsewhere [18]. This likely reflects the acute nature of the treatment schedule (i.e. completed by 48 h after each injury), thus limiting the time window during which the anti-CD11d mAb-treated rats might be more vulnerable to infection due to compromised leukocyte trafficking [18].

## Conclusions

Anti-CD11d mAb treatment reduced behavioral impairments, neuroinflammation, lipid peroxidation, neuronal loss, and axonal injury in the rat mLFP model of repeated concussion. At acute injury time-points, rats treated with anti-CD11d mAb at 2 h and 24 h after each of three repeated mLFP displayed significant reductions in unconsciousness and self-righting reflex times, cognitive impairments, neutrophil and macrophage levels, astrogliosis, lipid peroxidation, axonal injury, and neuronal loss compared to injured rats treated with a 1B7 control mAb. At the 8-week chronic injury time point, injured rats treated with the anti-CD11d mAb continued to exhibit reduced cognitive impairments as well as diminished neuroinflammation, and axonal injury relative to injured rats treated with 1B7 control mAb. In addition, the 1B7-treated rats performed significantly worse than sham-injured rats on behavioral tasks of anxiety-like behavior and sensorimotor function, whereas the anti-CD11d-treated rats did not. Although anti-CD11d-treated rats displayed some evidence of cognitive impairment, neuroinflammation, lipid peroxidation, neuronal loss, and axonal injury relative to sham-controls, they demonstrated significantly better outcomes compared to control antibody-treated rats. Taken together, these findings suggest that infiltrating leukocytes increase neuroinflammation, secondary brain damage, and functional impairment in a repeated concussion setting, and that the anti-CD11d mAb holds promise as a novel treatment to limit these effects.

## Abbreviations

ANOVA: Analysis of variance; APP: Amyloid precursor protein; CNS: Central nervous system; GFAP: Glial fibrillary acidic protein; LFP: Mild lateral fluid percussion injuries; LR: Long-recovery; LSD: Least significant difference; mAb: Monoclonal antibody; MDA: Malondialdehyde; PBS: Phosphate-buffered saline; SR: Short-recovery; TBARS: Thiobarbituric acid reactive substances; TBI: Traumatic brain injury; VCAM-1: Vascular cell adhesion molecule-1.

## Competing interests

The authors declare that they have no competing interests.

## Authors’ contributions

SRS carried out the behavioral studies and drafted most of the manuscript. FB carried out the biochemical and histological studies and drafted part of the manuscript. LCW contributed to data analysis and interpretation and to final editing of the manuscript. DPC headed the laboratory in which behavioral studies were done and supervised all aspects of these studies. AB headed the laboratory in which biochemical and histological studies were done, supervised all of those studies and wrote the final draft of the manuscript. All authors read and approved the final manuscript.

## Authors’ information

Sandy R Shultz and Feng Bao are co-first authors; Donald P Cain and Arthur Brown are co-senior authors.
